# Co-creating and hosting PxP: a conference about patient engagement in research for and by patient partners

**DOI:** 10.1186/s40900-024-00603-0

**Published:** 2024-07-29

**Authors:** Dawn P. Richards, Hetty Mulhall, Joletta Belton, Savia de Souza, Trudy Flynn, Alex Haagaard, Linda Hunter, Amy Price, Sara Riggare, Janice Tufte, Rosie Twomey, Karim M. Khan

**Affiliations:** 1 Canadian Institutes of Health Research Institute of Musculoskeletal Health and Arthritis, University of British Columbia, Vancouver, BC Canada; 2 Five02 Labs Inc., Toronto, ON Canada; 3 Patient Partner and Patient Author, Toronto, ON Canada; 4 Patient Partner and Patient Author, Fraser, CO USA; 5 Patient Partner and Patient Author, London, UK; 6 Patient Partner and Patient Author, Halifax, NS Canada; 7 Patient Partner and Patient Author, Kingston, ON Canada; 8 Patient Partner and Patient Author, Ottawa, ON Canada; 9 Patient Author, London, UK; 10 Dartmouth Institute for Health Policy and Clinical Practice (TDI), Geisel School of Medicine, Dartmouth College, Hanover, NH USA; 11 Patient Editor BMJ, London, UK; 12 Patient Partner and Patient Author, Stockholm, Sweden; 13 Participatory eHealth and Health Data, Uppsala University, Uppsala, Sweden; 14 Patient Partner and Patient Author, Seattle, WA USA; 15 PCORI, Seattle, WA USA

**Keywords:** Patient engagement in research, Patient and public involvement, Consumer involvement, Service user research, Patient-led conference, Co-production, PatientsIncluded, Patient author, Patient partner

## Abstract

Research projects, initiatives and conferences that include patients as partners rather than as participants are becoming more common. Including patients as partners (what we will call ‘patient partners’) is an approach called patient engagement or involvement in research, and we will call it patient engagement throughout this paper. Patient engagement moves traditional health research conferences and events to include a broader audience for their knowledge exchange and community building efforts, beyond academics and healthcare professionals. However, there are few examples of conferences where patients are given the opportunity to fully lead. Our conference went beyond patient engagement – it was patient-led. Patient partners conceived, planned, and decided on all aspects of a virtual conference.

We present the work and processes we undertook throughout 2023 to create and produce a free conference called “PxP: For patients, by patients” or PxP for short, with a tagline of “Partnering to make research stronger.” PxP was patient-led and about patient engagement in research rather than a specific disease or condition. PxP was supported by the Canadian Institutes of Health Research Institute of Musculoskeletal Health and Arthritis. The PxP website, known as the PxP Hub, now houses the conference recordings along with resources about patient engagement in research. These resources were recommended by the PxP Steering Committee members, speakers, and others who attended the 2023 conference. Here we lead you through how the idea for PxP was generated; how the international patient partner Steering Committee was convened and supported; how PxP was brought to life over nine months; the PxP 3-day event and feedback collected to improve future efforts; trade-offs, challenges and learnings; and resources required to support this type of event. We close with what the future holds for PxP in 2024 and beyond.

It’s time to elevate patients into leadership roles for conferences and events, and we encourage you to adopt the PxP ethos by using or adapting our approach and resources to support your opportunity.

**Supplementary Information:**

The online version contains supplementary material available at 10.1186/s40900-024-00603-0.

## Abstract (highlights)

Patients are often included in health research as study participants. Involving patients as partners in research projects or conferences is becoming more common. This approach is called patient engagement or involvement. Traditional health research conferences are by and for academic researchers or healthcare professionals. These events rarely include patients as the main attendees or in the planning. While some research conferences are starting to use patient engagement, few are designed and led by patients. We share our work on a conference led and designed by patients. In 2023, our team co-created a free conference called “PxP.” PxP is short for “For patients, by patients.” The conference tagline was “Partnering to make research stronger.” PxP was focused on patient engagement in research rather than any one health problem. PxP was supported by the Institute of Musculoskeletal Health and Arthritis. This is one of 13 Canadian Institutes of Health Research. A free online PxP Hub now hosts all of the conference recordings and many resources. We share how the idea for PxP came about, how the international patient partner Steering Committee came together and was supported to plan and bring PxP to life, the PxP conference and feedback, challenges and what we learned, and resources needed. It’s time for patients to have a leadership role for conferences and events. Through sharing this example, we encourage others to adopt the PxP ethos.

**Supplementary Information:**

The online version contains supplementary material available at 10.1186/s40900-024-00603-0.

## Background

While there are increasingly more research projects, initiatives and conferences that include patients as partners and not just participants, examples where patients fully lead are rare in these settings [1, 2]. To date, most of the focus in these spaces has been on co-creation or co-production aspects. The approach of including patients as partners (which we call ‘patient partners’) is referred to as patient engagement (in North America) [1], patient involvement (in Europe) [3], or consumer involvement (in Australia) [4]. Here we use the term patient engagement as a ‘catch all’ for these phrases.

Health research conferences and events provide an opportunity for knowledge exchange and community building, though have traditionally been restricted to a narrow audience of academics and health professionals. While some conferences are patient-led through conception, design, delivery and dissemination, these tend to be patient organization-run conferences that offer support and education to their specific communities (e.g., arthritis, pain, cancer, etc.) [5, 6]. To our knowledge, there is little in the academic literature about patient-led health research conferences [7, 8] and we were unable to locate any literature about patient-led conferences on the topic of patient engagement in research.

Here we describe the processes to plan and organize a free conference all about patient engagement in research called “PxP: For Patients, by Patients” and share the outputs of the conference [9]. The conference’s tagline was “Partnering to Make Research Stronger.” The free PxP conference was virtually hosted in September 2023, and was fully designed and driven by an international Steering Committee of individuals who identify as patient partners [1]. PxP was supported by the Canadian Institutes of Health Research (CIHR) Institute of Musculoskeletal Health and Arthritis (IMHA) [10]. IMHA’s mandate includes supporting research related to: active living, mobility and the wide range of conditions related to bones, joints, muscles, connective tissue, skin as well as the mouth, teeth and craniofacial region. IMHA has a history of engaging patients as partners for over 20 years [11]. Recently, IMHA and its Patient Engagement Research Ambassadors (PERA) fully co-created and launched free, online learning modules as a How-To Guide for Patient Engagement in Research which is ‘disease agnostic,’ meaning that it is not specific to any one disease area [12, 13]. PxP is another example of work that IMHA is supporting in the patient engagement in research space that is also disease agnostic.

This paper aims to describe what we did and learned following a planning and executing timeline so that people who are interested in elements of PxP may benefit when planning their own patient-led conferences or events. We detail the processes, provide templates and resources, and share our learnings by highlighting the expertise, time and other resources that were necessary to make PxP a success. In the spirit of PxP, this paper was co-written with many of its patient partner Steering Committee members (JB, SdS, TF, AH, LH, AP, SR, JT).

## Main text

Below we outline the PxP conference process, including its inputs and outputs, in a manner that is linear to PxP’s overall planning and executing timeline.

### Generating the idea for the PxP

The idea for the PxP conference came from conversations in 2022 with IMHA’s PERA members [12]. PERA is composed of individuals who live with conditions that fall under IMHA’s research mandate. PERA meets every 1–2 months virtually to provide bidirectional insights and lived experiences to help IMHA achieve its goals and priorities while also carrying out its own mandate. PERA’s mandate is to inform IMHA and CIHR of patient priorities in research; inform their respective communities about IMHA, CIHR, and their work with PERA; advocate to benchmark best practice in patient-oriented research (POR) across IMHA’s activities (including priority setting); curate quality POR assets (for example, videos, websites etc.) for IMHA and the broader CIHR community, and create new POR assets to fill gaps; and, evaluate progress of PERA [11]. PERA members who wished to contribute to the conference were invited to be part of the conference’s 2023 Steering Committee (SC; noting there were 6 PERA members at the time).

An initial discussion with interested PERA members (TF, LH, plus one other) was hosted in November 2022 to gain further insights. This initial conference discussion was supported by IMHA’s Scientific Director, a staff member, and a patient engagement consultant (DPR, who also identifies as a patient partner). Terms of reference for the SC were drafted (see Additional File 1) and a timeline of SC meetings with high-level agenda topics was created for internal IMHA purposes. The decision about the conference name was reserved for the patient-led SC. The only pre-determined parameters around the conference were that it would be: free, virtual, led by patient partners, about patient engagement in research, disease agnostic, and for patients (as a primary audience), though anyone would be welcome to attend (and those in academic roles heard about it through IMHA’s regular newsletter and other targeted newsletters). Patients being the primary audience was a decision purposefully made so that patients could explore topics about patient engagement that were most important to them in a safe space. After this meeting, in late 2022 and in early 2023, additional patient partners from across the globe were invited to be part of the SC (based on the diversity of their locations, experiences, etc.), with an aim to have a total of 10 members. SC members who were invited beyond PERA members were known to IMHA through various research and personal networks and social media interactions. One SC member left for personal reasons in spring 2023.

### Bringing the PxP conference to life

**Fig. 1:**
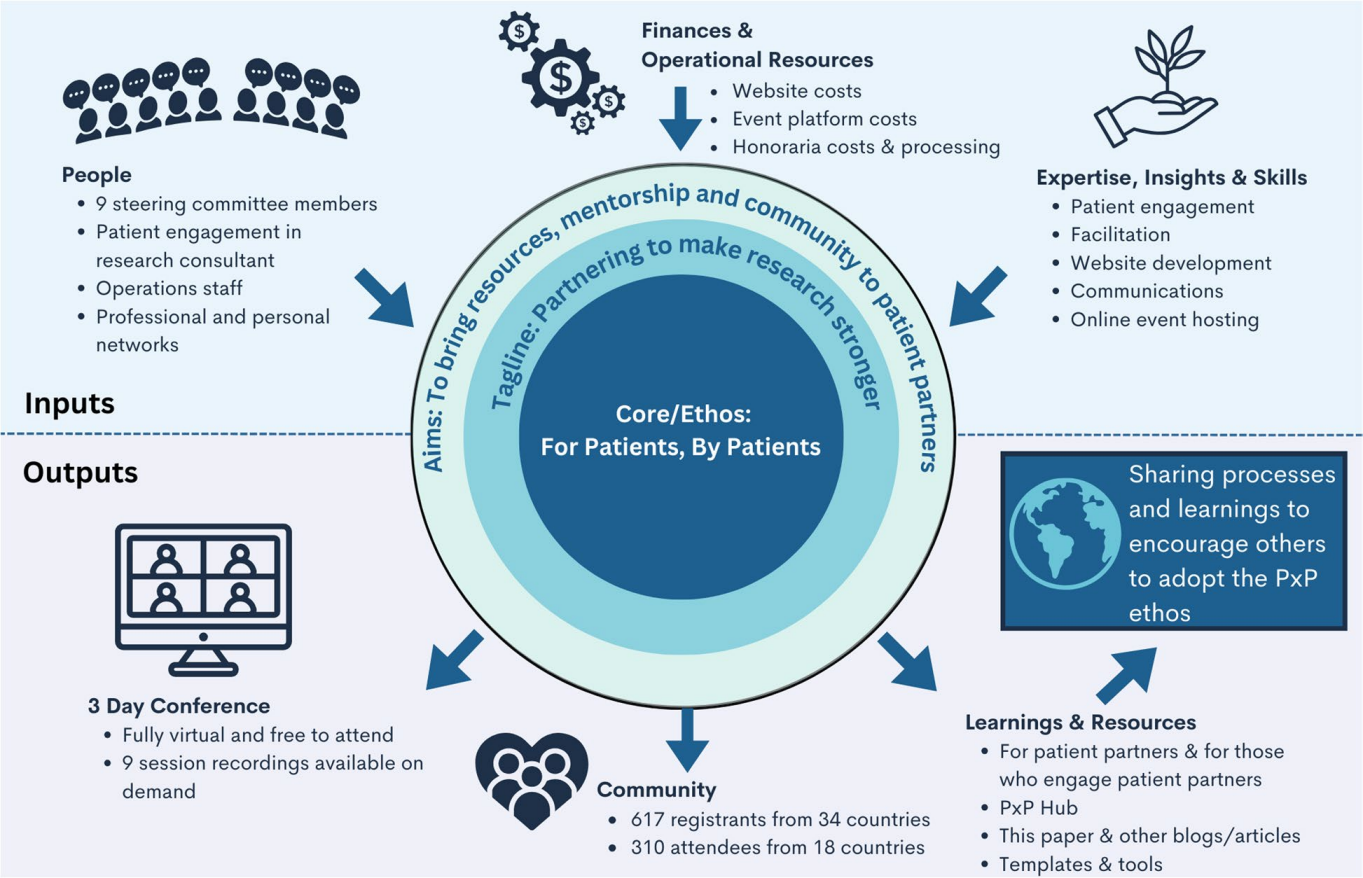
PxP Inputs and Outputs. An overview is provided of the human, financial and operational resources as well as the expertise, insights and skills that went in to PxP 2023. The various outputs include the 3-day conference, a community, and learning and resources

An overview of the inputs and outputs of PxP 2023 (see Fig. 1) are described in more detail in the following sections.

#### Championing the patient-led ethos

The international SC first met virtually in February 2023 and then monthly (except August and September) until October 2023 [14]. Two hours were reserved for each virtual meeting. The initial conference concept and parameters were brought to the SC during the February meeting so they could develop their collective vision. The development of the conference was guided by the five PatientsIncluded™ criteria [15], and went beyond patient co-creation to patient leadership. As outlined below, all elements of the conference were decided by the SC and facilitated through IMHA resources (time, financial, and skills).

The SC meetings were facilitated by the IMHA patient engagement consultant (DPR) and were attended by additional members of the IMHA team as needed to capture relevant action points to fulfill the logistical needs of the conference. DPR developed draft agendas for each meeting (which were open to changes made by SC members), took meeting notes (finalized upon review at each subsequent meeting) and facilitated meetings to ensure meetings supported psychological safety, respect, transparency and collaboration [16]. If SC members could not attend meetings, they were invited to provide their ideas separately via email or to meet one on one with DPR who incorporated their ideas in to planning. Meetings were kept to a minimum to be respectful of SC members’ time and other commitments. SC members were provided regular updates via email about operational progress between meetings. Email updates also included communication assets (such as social media graphics) so that the SC could share news about PxP with their own networks and on social media; with the opportunity to request additional assets, information or communication support at any point.

All SC members were offered honoraria aligned with the IMHA Patient Engagement Compensation Guidelines, which were developed after this work started [17]. Honoraria covered all aspects of SC members’ work and contributions for conference planning, and additional honoraria were offered for their time to participate in and attend the conference even if they chose not to be part of the program. Offering honoraria to SC members aligns with principles of equity, diversity and inclusion, and with best practices related to patient engagement in research [18].

#### Determining the path of PxP

Launching a new conference and an associated community required a significant amount of work and a focus on both the big picture and the more detailed elements (more on resources required is provided near the end of the paper). The patient-led and operations elements throughout the course of the PxP are detailed in Table 1. Over the course of conference planning, the SC decided on the following for the conference:

- its name (For Patients, By Patients) which was shortened to PxP;
- its goal of partnering to make research stronger;
- its mission to bring resources, mentorship and community to other patient partners in any kind of health research, no matter their experiences as a patient partner;
- its logo design and colours;
- the dates, format and length;
- its agenda, including each day’s themes, sub-themes and speakers;
- the platform (Zoom Webinars);
- the preferred communications channels (X/Twitter, LinkedIn, Instagram and newsletter); and,
- the post-conference survey questions for attendees, hosts, moderators, and speakers.

**Table 1:**
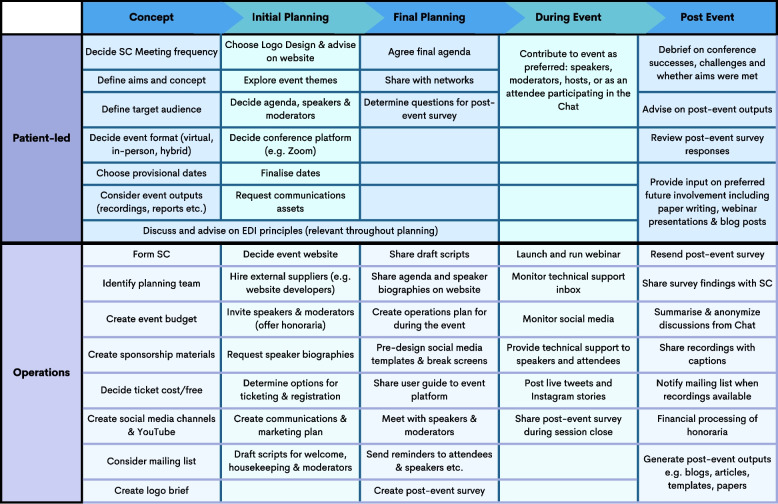
Patient-led and operations elements of PxP

The table denotes patient-led and operations elements of PxP throughout the various phases of PxP (from the concept to initial and final planning, through to during and post-event)

Additionally, the SC advised on the PxP website (also called the PxP Hub) [19] by providing thoughts on: user journey, functionality, content and accessibility features. Each member also provided up to 3 resources about patient engagement in research that are posted on the site. The PxP website was created by an agency and is updated by an IMHA staff member.

#### Co-developing the program

The themes for each day of the conference and for the sessions themselves were selected based on several SC conversations at the monthly meetings. The conference agenda including each day’s theme and sessions is provided in Table 2, with the full conference agenda that includes dates, times, hosts, moderators, and speakers/panelists in Additional File 2. Day 1 was considered to be introductory to help equip attendees with the language and baseline knowledge necessary to get involved in patient and public engagement in research, as well as to get the most out of the program [20]. Day 2 was designed to highlight self-research (where speakers shared doing their own research on themselves as part of their own healthcare journeys), patient-led research, and researcher perspectives on the how and why of their patient engaged research [20]. On Day 3, the SC wanted to tackle difficult conversations around real-world challenges for patient partners and those who have historically been excluded from health research in the safe space of this patient-led conference, and finished with a session providing practical tips around knowledge dissemination and amplifying the impact of health research [20]. The themes and session topics were of importance to the SC as patient partners rather than those researchers thought would be important to them.

**Table 2:**
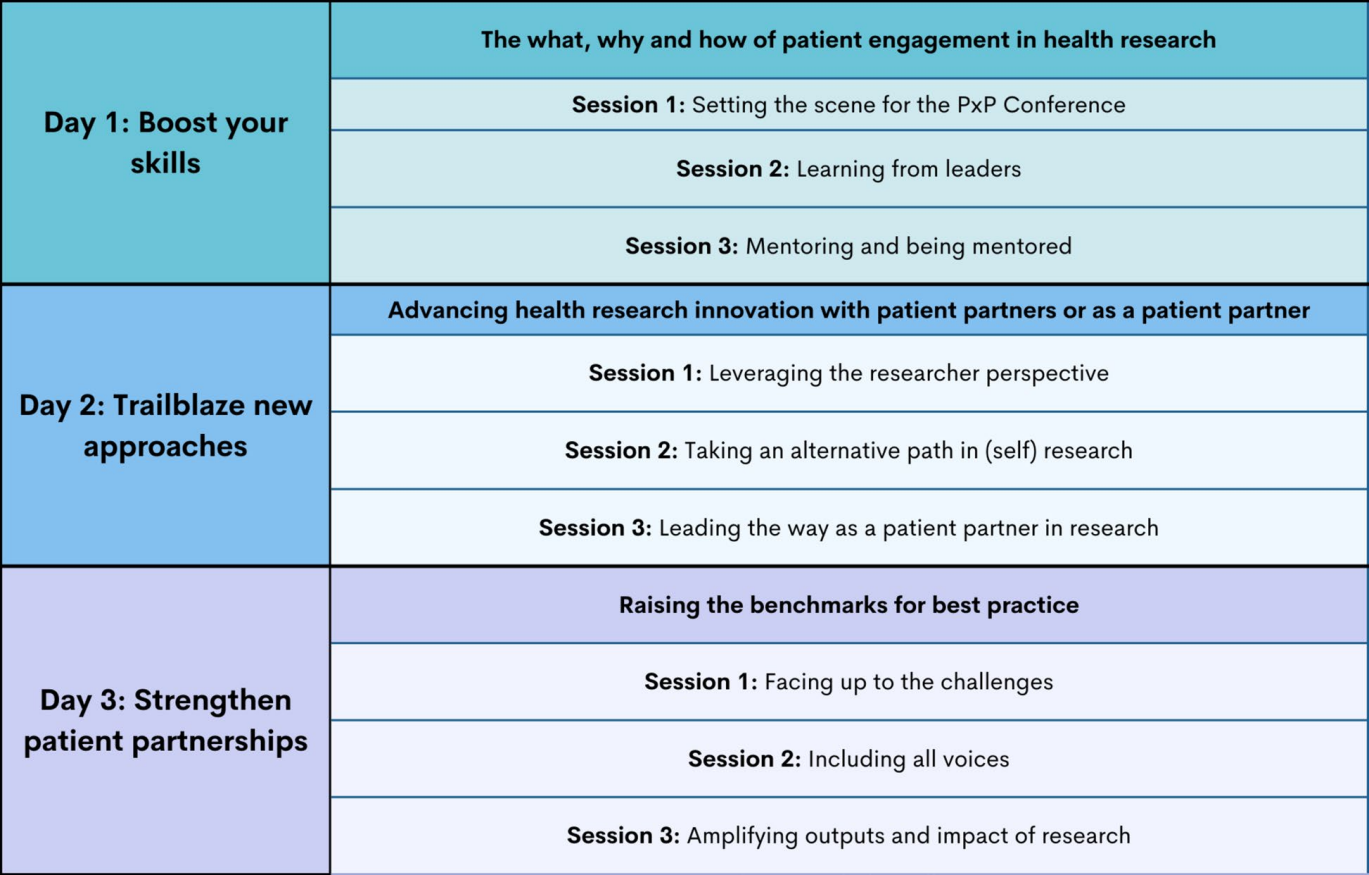
PxP conference agenda, including each day's theme and corresponding sessions

The table shares the PxP agenda for the 2023 conference. Each day had an overall theme with a brief descriptor, and each of the day's sessions had titles tying in to the overall theme for the day

All SC members had multiple opportunities to suggest specific people or organizations as speakers. They also had the opportunity to be as involved or uninvolved as they wished with the program itself, including as hosts (opened and closed each day and handed each session over to the session moderator), moderators (facilitated sessions by introducing the topic, speakers, and asking audience questions), and speakers, or, if time-zones permitted, as attendees who contributed to the live chats and encouraged discussion amongst attendees. SC members also contributed to how each conference session would be structured, being purposeful about having different approaches to each session (some included presentations, most were moderated discussions). DPR collected, compiled and reviewed all suggestions, and then proposed a draft agenda with a number of options for speakers, which took in to account equity, diversity, and inclusion (EDI) principles [16]. This agenda was subject to additional discussion, changes, and then approval by the SC before speakers were invited.

#### Conference supports

SC members who participated as hosts, moderators, and speakers/panelists were supported by the IMHA staff and DPR, as were all other moderators and speakers/panelists. All hosts, moderators and speakers/panelists were offered monetary compensation for their time in preparing for and being involved in the conference, with the exception of those who had an academic appointment and whose involvement in such a conference was considered part of their role in academia. For each session of the conference, a meeting was set up and facilitated by DPR so the moderator and speakers/panelists of that session could meet and get to know one another in the month leading up to PxP. At these virtual meetings, logistics about the conference were reviewed and notes were taken that were provided back to attendees. In most cases, the participants worked together to co-create the session content (often with suggestions from SC members).

A ‘run of show’ was created in Google Docs and was shared with all SC members for comment. The ‘run of show’ included each day’s schedule, logistics comments for daily hosts to share with all PxP attendees at the start and close of each day, transitions to breaks, and notes for each session about how it would run, who the moderator and speakers were, etc. The daily hosts were encouraged to make the logistics comments and all commentary their own by copying and pasting content out of the Google Doc. The ‘run of show’ for each session was shared with each session’s moderator, speakers or panelists. Two drop-in sessions on different times and days (to accommodate a variety of time-zones) were offered to all session speakers or panelists and hosts the week before the conference as an opportunity to gain familiarity with the platform, ask logistical questions, and test out slide sharing functionality. Communications to moderators and speakers and panelists were intentionally minimized, but carefully crafted to ensure language was clear and support was available if needed. Formal and informal feedback indicated that hosts, moderators and speakers felt well-prepared for and supported at the conference.

#### Being purposeful about accessibility

The SC was clear about the importance of accessibility, that is removing barriers to attend and fully participate, being mindful that the conference audience would include people with a wide range of disabilities and access needs, including energy-limiting chronic illness and neurodivergence. Registrants were invited to contact the IMHA team to make it aware of any access or accommodation needs that were not being provided so that it could do its best to support them. The conference was free and the program was purposefully designed with 30-min breaks between sessions. The timings of the program were intended to suit people joining from different time-zones around the world, with Day 3 run during different hours to better include attendees and speakers in Oceania and parts of Asia. To help reduce the burden of converting between time-zones, a table was provided on the PxP website [21].

As an ongoing consideration to accessibility and inclusivity, all sessions were recorded (with consent) and are available to view directly on the PxP YouTube Channel [22] or via the PxP Hub [9]. Asynchronous viewing was considered important for those who could not join live (for example, due to time-zone, medical needs, or other responsibilities) or who would like to re-watch the sessions. The entire transcript was manually edited with Adobe Premiere Pro to help improve accuracy of closed captions. The Adobe software was available through an institutional subscription and there may be alternative options for those who do have access to this product. Anonymized chat summaries from the live sessions are also available for each day on the PxP Hub resource page.

Accessibility for all participants was a priority with the logo and website design, and highlighted in the logo brief and website scope of work. The logo and website have sufficient colour contrast to meet Web-Content Accessibility Guidelines and the colour palette was chosen to not be visually overwhelming for people with visual processing challenges [23]. The decision to exclude icons was purposeful; different images may not translate well between patient communities and across different cultures. An easy-to-read font was chosen. The premium version of UserWay was added to the site which includes a range of tools for users such as the ability to adjust font, line height or contrast; to pause animations; and to utilise a screen reader, reading mask or reading guide [24]. There can be limitations and problems that may arise from using automated accessibility overlays which should be investigated and understood before using one. A web page to explain these features and signpost to contact details for additional support is featured prominently on the main menu of the website [25]. The website is bilingual, offered in both of Canada’s official languages, English and French. Speaker biographies were provided in three different options, in recognition of different preferences or needs for content format: html, PDF with selectable text, and digital flipbook optimized for mobile devices [26].

The SC chose Zoom Webinars as the conference platform to help promote inclusivity given the familiarity that many people have with it. To keep things simple with the first PxP, a conscious decision was made to use Webinar without any breakout rooms or networking sessions. Attendee and speaker/panelist versions of a PxP 2023 Zoom Webinars guide were created with images to help people get set up at the conference [27]. See Additional File 3 for the attendee Zoom Webinar guide. A member of the IMHA logistics team was available throughout the conference as technical support for attendees, and a second Zoom room was set up for speaker/panelist questions. At the start of each day of the conference, the daily host provided information to attendees about supports provided and how to access them (e.g., closed captions for sessions, etc.). A Zoom Webinars background was automatically provided for speakers/panelists when they logged in to the webinar, and the colour was chosen to reduce brightness for those with visual processing needs. The logistics team ensured the Zoom platform had the live translated captions as an add-on in over 30 languages. While the addition of live interpreters in other languages including sign-language is preferred, this was not viable due to the number of countries and languages represented in the audience, the conference budget, and other resource considerations.

### PxP live and feedback

#### PxP live

PxP 2023 took place virtually on September 12, 13 and 14/15, 2023 UTC over a total of 4 h (including breaks) on each day (see Additional File 2 for the full agenda). There were a number of powerful quotes as takeaways from the sessions, some of which are provided in Table 3. PxP 2023 had 617 registrants from 34 countries, with 310 live attendees self-identifying that they were from 18 countries (note SC members and IMHA staff are included in this number). Attendees were from around the globe including from North America (Canada, United States), South America (Brazil, Colombia), Europe (Denmark, France, Ireland, Italy, Portugal, Romania, Spain, Sweden, United Kingdom), Asia (India, Israel), Africa (Nigeria), and Oceania (Australia, New Zealand). The majority of attendees were from Canada (57%), United States (18%), United Kingdom (11%) and Australia (7%), respectively. Attendees were provided the option at the start of each day to identify the perspective they brought in a Zoom poll (they were allowed to select more than one option), and in these polls (data are summarized across all 3 days), attendees identified themselves as a patient partner/person with lived experience (59%), a researcher (35%), a caregiver or relative (20%), a clinician (9%), or a trainee or student (6%).

**Table 3:**
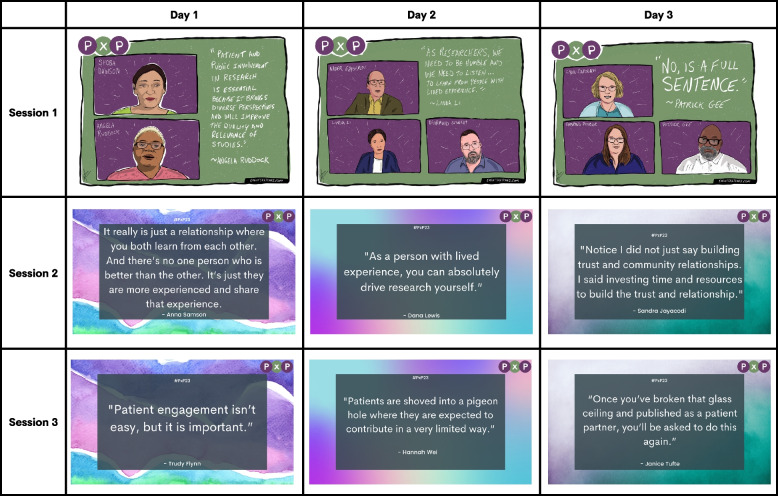
Quotes or takeaways from each session of PxP 2023

The table shares a quote from each session on each day of PxP and is attributed to a participant in each session. The top row includes sketches of speakers done live by a graphic artist in attendance

One member of the IMHA team hosted Zoom Webinars and DPR was also on the ‘back-end’ of Zoom Webinars to support each session’s participants if/as required. SC members took on a variety of roles including as hosts, moderators, presenters, or active participants watching and participating in the Zoom chats. In addition to housekeeping, a Land Acknowledgement [28], and a bit of information about the day, daily hosts encouraged engagement from the start by asking the audience to respond to the Zoom poll mentioned above.

A graphic artist sketched each speaker/panelist in each day’s first session along with one of their quotes from the session. Once these sketches were signed off by the sketched speakers/panelists, they were shared on social media. There are plans to build on this idea for the 2024 conference by engaging an artist who identifies as a patient to do this for all sessions of the conference.

#### Attendee interactions and social media

Before the conference, all individuals who registered were provided with a link to a guide to make the most of Zoom Webinars and encouraged to share anything they wished in the chat and on social media with the hashtag #PxP23. At the start of each day, hosts provided introductory comments that included a short overview on using Zoom’s chat, question and answer (Q&A), and emoji functions, the latter to provide live feedback to hosts, speakers and moderators. These appeared to be successful tactics for audience participation as there was a great deal of commenting and sharing of resources in the chat, of emojis during presentations and conversations, and efforts made to include questions from the audience into each session (at the end for a dedicated question and answer session or throughout if the session was a moderated discussion). Since permission was not sought of attendees to copy and share the chats verbatim, two IMHA staff created anonymous summaries of each session’s chat comments and resources that were shared in the chat, which are available on PxP Hub in its resources section. [29].

Leading up to PxP, a newsletter and social media handles were created on Twitter/X (@PxPHub), Instagram (PxPHub), Threads (PxPHub), and LinkedIn (PxPHub). On the days of the event, PxP live updates and threads were shared primarily on X, in addition to Instagram Stories and LinkedIn. A social media graphic was prepared in advance to be used for event quotes and all images were shared with Alt text. In September 2023 (the month of the conference), the PxP X account gained 94.5 k organic (i.e., not paid for) impressions with a 2.4% engagement rate; on PxP LinkedIn, organic impressions were 3,859 with a 12.2% average engagement rate (which is the engagement rate for each post divided by the total number of posts). The newsletter has over 700 subscribers with an average open rate of 57% and an average click rate of 10%.

Between its launch on July 19, 2023, and December 31, 2023, the PxP website had 5.9 k unique visitors, 16.3 k page views, and an average visit duration of over 2 min. The top four countries for website views were Canada (46%), United States (20.9%), United Kingdom (12.3%) and Australia (9.5%), which mirrors the geographic makeup of PxP attendees. Most people were accessing the site directly (for example, through email share), followed by through X, Google, LinkedIn and Facebook. Other than the homepage, the most frequently accessed pages related to the event tickets and program and the PxP resource page, which is a collation of PxP and external resources.

All PxP 2023 session recordings were made available to view on the PxP YouTube Channel [22] following manual editing of the closed captions. Day 1 recordings were shared in September 2023, Day 2 recordings in October 2023, and Day 3 recordings in November 2023. As of December 31, 2023, the recordings have already garnered 546 views from over 200 unique viewers, with a combined watch time of 90.5 hours; the videos also have 2 k impressions (which is the times the video thumbnails were shown on YouTube) and an impressions click-through rate of 4.8%, this includes people who have been shown the content in their suggested videos, or Browse features (for example).

#### Conference feedback

A Project Ethics Community Consensus Initiative (ARECCI) framework was used to assess for and mitigate ethical risks for a survey of PxP participants (hosts, speakers, moderators, and attendees), including the four-step ARECCI Ethics Screening Tool and the ARECCI Ethics Guidelines [30, 31]. The survey was deemed as minimal risk to participants and did not require review from a Research Ethics Board. All PxP participants (hosts, speakers/panelists, moderators and attendees) were provided a link to an online, voluntary, self-reported anonymous survey to complete. The survey included a consent statement at the beginning about the potential use of their results and open-ended responses for learning purposes or for publication purposes. Respondents could opt-out of their survey responses being used for publication purposes if they wished. The survey was issued using a modified Dillman’s method to achieve a better response rate [32].

All 310 attendees were invited to respond to a survey about the conference. One-hundred and thirty-seven (137) attendees voluntarily submitted survey responses (response rate of 44%). Of these respondents, 136 respondents consented to their responses being used for publication purposes and 1 respondent agreed for their responses only to be used for learning purposes, not for publication purposes. The survey results from the 136 attendees were overwhelmingly positive:

- 96% (130) of respondents agreed or strongly agreed that the themes and topics discussed were relevant to them;
- 96% (130) of respondents agreed or strongly agreed the question and discussion periods were well-organized and helpful in their learning;
- 74% (100) agreed or strongly agreed that there were opportunities to interact, engage and network with other attendees during the conference;
- 88% (120) of respondents agreed or strongly agreed they learned something new that will be useful in their future approach in patient partnership/engagement;
- 95% (129) of respondents agreed or strongly agreed that the conference environment was inclusive and safe;
- 95% (129) of respondents agreed or strongly agreed that they would recommend the PxP conference to a friend or colleague;
- 95% (129) of respondents agreed or strongly agreed that they were satisfied with Zoom Webinars as the conference platform; and,
- 94% (128) of respondents agreed or strongly agreed that they were satisfied with the sessions they attended.

In response to 5 comments on main areas for improvement, increased diversity (gender, ethnicity, geography) for SC members and speakers will be a focus for PxP2024.

SC members were also issued a separate online survey based on the validated Patient and Public Engagement Evaluation Tool to respond to anonymously about their experiences of being a SC member between February to September 2023 [33]. This survey also had a consent statement at the start about potential uses of the survey data and from which respondents could opt-out. All 9 SC members responded anonymously to this survey. The results indicated that their experiences were positive overall with respect to planning and carrying out the conference. Overall, they indicated that the SC was a safe environment where they could express their views, they felt supported by IMHA in a number of ways (e.g., through being offered one on one meetings if they were unable to attend scheduled meetings), they felt that their feedback was taken into account by IMHA, and that they were proud of the conference. Like conference attendees, they felt that the SC’s diversity of experiences, ethnicity, gender, geography, etc., should be expanded in future years.

### Trade-offs, challenges and learnings

The SC aimed to minimize the burden of registering for and attending the conference. The SC decided a conference platform was not necessary, and instead opted to use Zoom alone given familiarity with Zoom due to the COVID-19 pandemic. Further, the SC considered the options of separate registrations and links for each session vs. each day of the conference, and opted to have each day run as one long Zoom Webinar. It was thought that this registration/link approach would minimize potential for confusion around different time-zones for an international event. With this approach, each attendee registered for each day and was provided with a personalized link for that day. Using Zoom Webinar meant that for the starting session of each day, session hosts and speakers/panelists were in a private Zoom Webinar room before the event went live and could prepare without the audience seeing or hearing them. However, after that first session and during breaks, new session speakers/panelists joined and if audience members kept their Zoom Webinar open, they were privy to these preparations. Using Zoom Webinar also meant that attendees could not direct message each other, rather only had the option to post to everyone in the chat. Visual messaging on the screen and in the chats was used during the breaks to let attendees know when sessions would start again. Even with these trade-offs, evaluation results and comments indicated that Zoom Webinar was an appropriate platform for PxP 2023.

Some audience members found the action in the Zoom Webinar chat during the sessions to be distracting and struggled to keep up with the amount of participation in the chat. Part of this may have had to do with how their chat settings were set up (some participants indicated that the chat kept ‘popping up’). For PxP 2024, some suggestions will be offered to deal with the chat and how it can be minimized, and attendees will be informed that the chats will be summarized (including the resources shared in the chats) and posted on the PxP resource hub after the conference.

Future conferences will see an increased diversity (gender, ethnicity, etc.) of the SC members and the invited speakers. Four survey comments indicated gender diversity is an area for improvement given they observed few who appeared to identify as men presenting in the sessions (note that organizers did not ask hosts, moderators or speakers/panelists to disclose their gender). A study of the demographics of patient partners in Canada indicates that this gender uniformity is fairly reflective of the patient partner demographic in many initiatives [34].

Even though this event was all about patient engagement in research and aimed to execute well on best practices, we experienced process challenges at IMHA’s home institution with respect to issuing honoraria for international participants. The IMHA team has developed an approach to minimize these process issues by working with its home institution for subsequent events.

### Resource requirements

The conference required certain resources from IMHA. In addition to a financial budget, a conference platform, communication tools (e.g., a website, social media accounts, a newsletter, etc.), and human resources were required. Without these resources, it would have been difficult to host the same quality of PxP.

It is estimated that in addition to IMHA human resource costs (see below for information on time of various roles), the cost for the first PxP was approximately $27,500 CDN (all figures here in Canadian dollars). This amount includes honoraria offered to SC members (to attend meetings and for various roles in the conference) and all speakers/panelists ($20,000), building the website ($6,000, excluding an annual maintenance fee of $900), and the Zoom platform (~ $1,500). For PxP2024, the only item that will come off the budget is the cost to build the website.

While time commitment varied and especially ramped up closer to the conference itself, a number of IMHA team members contributed to the conference. The financial cost of these human resources is not provided as a dollar amount as this will vary for organizations. Balancing other IMHA-related work within her one-day a week commitment to IMHA (that is, less than 0.2 full time equivalents), DPR prepared materials for and hosted all SC meetings, met with SC members individually if they could not attend scheduled meetings, invited speakers and panelists, hosted 9 conference planning sessions/introductory meetings for sessions, attended the 2 pre-conference drop-ins for all speakers, hosts, and panelists, led developing the ‘run of show,’ shared information about the conference on social media and in her networks, and supported speakers, hosts and panelists on the back-end of Zoom Webinars each day of the conference. RT worked closely with DPR and attended all SC meetings and provided technical support for speakers, hosts and panelists at the conference. HM worked closely with DPR, attending most SC meetings, co-designed all communications and social media assets for the conference, coordinated all IMHA communications about the conference, coordinated PxP logo and website development, created all conference agendas and guides for using Zoom Webinar, etc., and hosted Zoom Webinar for the conference. HM’s time commitment was between 0.25–0.5 full time equivalents in the 6 months leading up to the conference. Another member of the IMHA team supported compensation processes for all SC members, speakers and panelists, through setting up individuals with a finance system and ensuring payments were received. And one other member of the IMHA team co-created the evaluation materials and uploaded them in to an online survey software (Qualtrics), and helped with analyzing all survey results. KK supported the entire project by attending SC meetings, the conference itself, and allowing IMHA resources to be used to support the PxP.

### Next steps for PxP and beyond

Planning for PxP 2024 has already started, and PxP will continue until at least 2025 with IMHA support. Beyond 2025, a new Scientific Director will be appointed to IMHA (Scientific Directors’ terms are for a maximum of 8 years, with the current Scientific Director completing his term in 2025), and their support for PxP is not guaranteed. With a commitment to provide new patient partners the opportunity to plan and participate in the conference, a new SC is being formed for 2024 with efforts to expand its diversity (patient partner experience, gender, ethnicity, geography, etc.), and based on suggestions made by 2023’s SC members. The 2023 SC has become an Alumnus Committee and their interaction with the 2024 SC and involvement in PxP 2024 will be decided by the incoming SC. As was the case with the 2023 event, the 2024 SC will decide on all aspects of the conference, building on the successes and learning from the challenges of PxP 2023.

As individuals who were involved in designing and executing PxP, we encourage others to use the PxP template and build on it to find ways to support and create conferences and events for patients and by patients. Support may take the form of any of a number of resources, such as people, funding, and skills. One of the main challenges for patients planning and carrying out their own conferences is funding, and we urge organizations to consider how they can provide this type of financial support. There may also be more innovative partnership models waiting to be created and learned from.

## Conclusions

We present work we undertook throughout 2023 to co-create and produce PxP, a conference for patients and by patients, all about patient engagement in research, and its associated PxP Hub. We share how the conference prioritized the patient partner community and accessibility, and provided an opportunity for knowledge exchange and nuanced discussions on all aspects of patient engagement in research. The conference has convened an active community of over 700 people that we hope will grow. We aim to engage in future events, with a deeper focus on equity, diversity and inclusion. In addition to working with knowledgeable patients who have their own networks, having support (funding, project management and communications skills and expertise, people, etc.) is required to carry out such an event. The time is now to elevate patients into leadership roles for conferences and events. We encourage you to adopt the PxP ethos by using or adapting our approach and resources to support this model.

## Supplementary Information

Additional file 1. Steering Committee Terms of Reference.

Additional file 2. Full version of the conference agenda which includes dates, times, hosts, session names and their respective moderators and speakers.

Additional file 3. Zoom Webinar Guide that was created for conference attendees to make the most of attending the conference on the Zoom Webinar platform.

## Data Availability

No datasets were generated or analysed during the current study.

## Abbreviations

CIHR: Canadian Institutes of Health Research
IMHA: Institute of Musculoskeletal Health and Arthritis
PERA: Patient Engagement in Research Ambassadors
POR: Patient-Oriented Research
PxP: For Patients, by Patients
SC: Steering Committee
