# Correction: Co-creating and hosting PxP: a conference about patient engagement in research for and by patient partners

**DOI:** 10.1186/s40900-025-00671-w

**Published:** 2025-01-23

**Authors:** Dawn P. Richards, Hetty Mulhall, Joletta Belton, Savia de Souza, Trudy Flynn, Alex Haagaard, Linda Hunter, Amy Price, Sara Riggare, Janice Tufte, Rosie Twomey, Karim M. Khan

**Affiliations:** 1 Canadian Institutes of Health Research Institute of Musculoskeletal Health and Arthritis, University of British Columbia, Vancouver, BC Canada; 2 Five02 Labs Inc., Toronto, ON Canada; 3 Patient Partner and Patient Author, Toronto, ON Canada; 4 Patient Partner and Patient Author, Fraser, CO USA; 5 Patient Partner and Patient Author, London, UK; 6 Patient Partner and Patient Author, Halifax, NS Canada; 7 Patient Partner and Patient Author, Kingston, ON Canada; 8 Patient Partner and Patient Author, Ottawa, ON Canada; 9 Patient Author, London, UK; 10 Dartmouth Institute for Health Policy and Clinical Practice (TDI), Geisel School of Medicine, Dartmouth College, Hanover, NH USA; 11 Patient Editor BMJ, London, UK; 12 Patient Partner and Patient Author, Stockholm, Sweden; 13 Participatory eHealth and Health Data, Uppsala University, Uppsala, Sweden; 14 Patient Partner and Patient Author, Seattle, WA USA; 15 PCORI, Seattle, WA USA

**Correction: Research Involvement and Engagement (2024) 10:77** 10.1186/s40900-024-00603-0

Following publication of the original article [1], the authors identified an error with Fig. 1. The correct figure is given below.

The incorrect Fig. 1 reads:
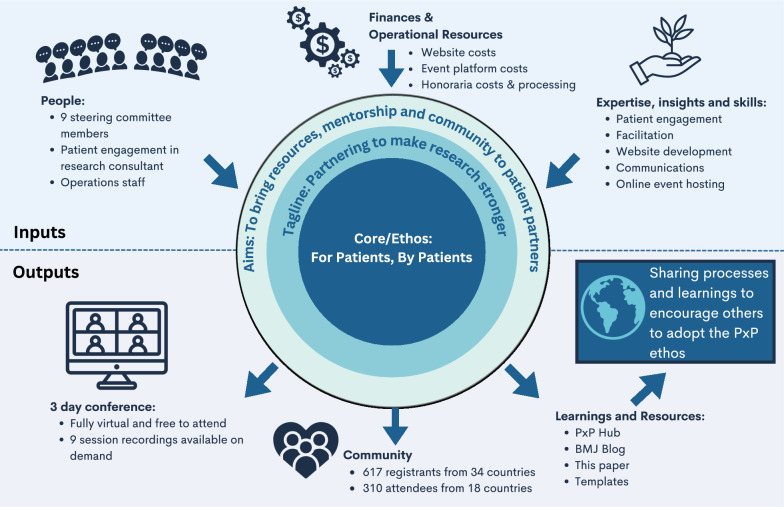
**Fig. 1** PxP Inputs and Outputs. An overview is provided of the human, financial and operational resources as well as the expertise, insights and skills that went in to PxP 2023. The various outputs include the 3-day conference, a community, and learning and resources

Figure 1 should read:
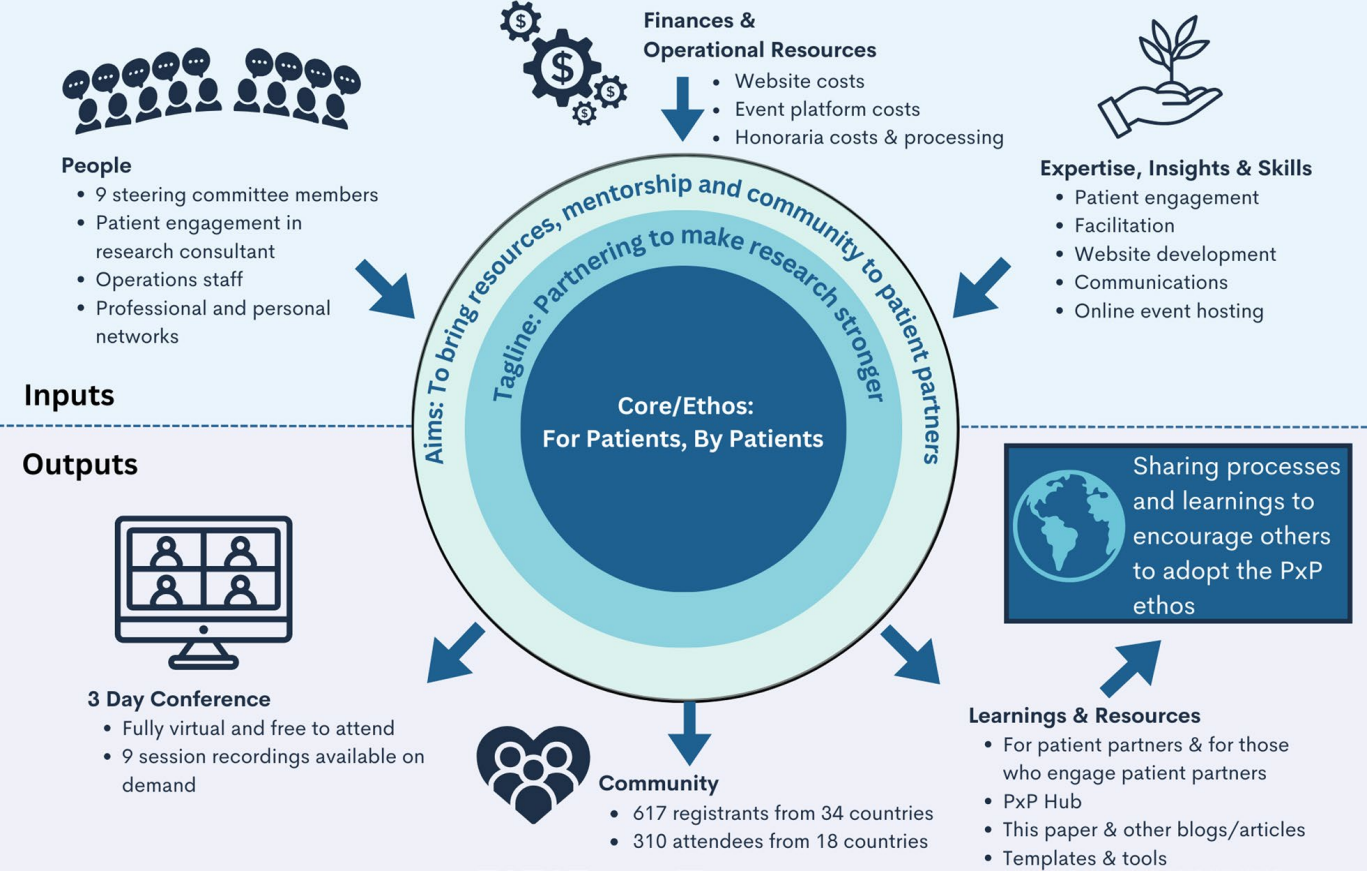
**Fig. 1** PxP Inputs and Outputs. An overview is provided of the human, financial and operational resources as well as the expertise, insights and skills that went in to PxP 2023. The various outputs include the 3-day conference, a community, and learning and resources

Figure 1 is implemented in this correction and the original article [1] has been corrected.
