# Supplementary material for: Co-creating and hosting PxP: a conference about patient engagement in research for and by patient partners
Source: Res Involv Engagem. 2024 Jul 29;10:77. doi: 10.1186/s40900-024-00603-0 (PMC11287933; doi:10.1186/s40900-024-00603-0)
Supplement: Supplementary file 1 — Additional file 1. Steering Committee Terms of Reference. [file 40900_2024_603_MOESM1_ESM.pdf]

CIHR Institute of Musculoskeletal Health and Arthritis
Terms of Reference: Patient Engagement in Research Conference – By and for Patients

Introduction

The Canadian Institutes of Health Research (CIHR) is Canada's federal funding agency for health research. CIHR is made up of 13 Institutes — communities of experts in specific areas who collectively support a broad spectrum of biomedical, clinical, and health systems research, and the social, cultural and environmental factors that affect the health of Canadians. CIHR collaborates with partners and researchers to support the discoveries and innovations that improve our health and strengthen our health care system.

The Canadian Institutes of Health Research (CIHR) Institute of Musculoskeletal Health and Arthritis (IMHA) is mandated to provide research leadership related to: active living, mobility and the wide range of conditions related to bones, joints, muscles, connective tissue, skin as well as the mouth, teeth and craniofacial region. Musculoskeletal health is critical for mobility, productivity, and general well-being. The institute has actively encouraged the research community to embrace incorporating patient engagement in the research process since its inception in 2000 and more recently is providing the resources to do it. While IMHA co-creates patient engagement resources with patient partners, it recognizes that many aspects of patient engagement can be disease- agnostic. The How-to Guide to Patient Engagement in Research modules released in fall 2022, exemplifies how IMHA strives to help the entire research community with respect to patient engagement.

In this spirit, IMHA seeks to develop a conference on patient engagement in research by and for patients (an overarching term that includes individuals with lived experience, including patients, caregivers, family, and friends[1]). The first of these annual events will be held in September 2023. The conference will be planned and hosted by patients, and this work will be facilitated and supported by IMHA. The primary audience is patients. We are confident the conference will also be of interest to others involved in patient engagement. The conference will be virtual and aim for international speakers and participants.

Purpose of this Document

This Terms of Reference document describes the goals, composition, and responsibilities of the conference's Steering Committee. It also describes the support that IMHA will provide to the Steering Committee.

Steering Committee Goals

The overall conference theme is patient engagement in research, with the aim to explore patient engagement across the full spectrum of research. The Steering Committee will be responsible for planning the conference including the setting the conference objectives, format,

[1] CIHR's definition of patient, http://cihr-irsc.gc.ca/e/48413.html

sub-themes, program and identifying speakers. Members of the Steering Committee should also expect to attend the conference and may be involved, to the extent to which they are comfortable, in its execution by taking on various roles (e.g., panel host or moderator, conference facilitator, speaker, etc.). They may also be asked to provide introductions/contact information for any potential speakers/other resources to whom they are connected.

Steering Committee Membership
The Steering Committee will be comprised of 8-10 patients who have experience in patient engagement in research. The Committee members will be invited from diverse geographic locations, and have different conditions, identities, communities and experiences. The approach intends to ensure many voices are heard, amplified, and reflected in conference programming and so the conference may appeal to a wide audience.

Steering Committee Responsibilities
The Steering Committee members are asked to:

- Arrive prepared to contribute to meeting discussions, having read any meeting materials in advance
- Respond to (minimal) emails (or Slack channel communications) between meetings about the conference (e.g., those that are time sensitive or require a decision between meetings)
- Respect confidentiality of meeting conversations and information that others share about themselves
- Engage in respectful conversation, even when in disagreement with others
- Ask questions if unsure or unclear about expectations or discussions
- Make decisions by consensus (agreement by the majority with minority objections as a result of discussion)
- Share approved materials about the conference within their own networks
- Participate in the conference as they are comfortable and able to (e.g., as an attendee, speaker or as a facilitator/moderator of a session).

Meetings and Operations
Meetings will be organized once a month between January and August 2023, and it is anticipated that each meeting will be 2 hours. The meetings will be organized at times/dates that work for most Steering Committee members (these will be scheduled in January/February 2023, with the intent to have everyone join all meetings, if possible).

IMHA will provide support to the Steering Committee, including facilitators for the meetings. The Steering Committee facilitators are Drs. Dawn Richards and Rosie Twomey. Their responsibilities will be to:

- Create a safe space for all Steering Committee members to engage
- Organize and chair meetings

- Establish and communicate meeting agendas, with an aim to send materials out one week in advance of meetings
- Facilitate meetings and ensure all Steering Committee members are heard
- Take notes (including highlighting action items) for each meeting and circulate these
- Ensure Steering Committee members who are unable to make a meeting have provided input to that meeting's discussion and decisions in other ways if they wish to (e.g., via email or separate phone/video-call)
- Ensure follow through on actions from meetings
- Ensure the Steering Committee fulfils its purpose.

Remuneration

Members of the Steering Committee will receive an honorarium for their participation in the planning process and the conference should they wish to receive it. The amount will be confirmed and communicated to Steering Committee members at the first meeting in February 2023. It is the responsibility of individuals who accept the honorarium to understand any financial implications associated with acceptance of an honorarium.

Version Control

Version	Notes	Date
Drafted	Initial draft for discussion at first Steering Committee meeting.	February 2, 2023
