# Supplementary material for: Co-creating and hosting PxP: a conference about patient engagement in research for and by patient partners
Source: Res Involv Engagem. 2024 Jul 29;10:77. doi: 10.1186/s40900-024-00603-0 (PMC11287933; doi:10.1186/s40900-024-00603-0)
Supplement: Supplementary file 2 — Additional file 2. Full version of the conference agenda which includes dates, times, hosts, session names and their respective moderators and speakers. [file 40900_2024_603_MOESM2_ESM.pdf]

PxP 2023

DAY 1: BOOST YOUR SKILLS

The what, why and how of patient engagement in health research.

September 12 (UTC)

Time (UTC)	Session	Speakers
**4:00 pm - 4:10 pm**	**Conference Welcome**	Day one host:<br>Amy Price
**4:10 pm - 5:00 pm**	**Session 1 - Opening Talk**<br><br>**Setting the Scene for the PxP Conference:** an overview from a patient partner and a researcher.	Angela Ruddock and Shoba Dawson<br><br>Building sustainable partnerships: A public contributor and researcher perspective<br><br>**Moderator:** Amy Price
**5:00 pm - 5:30 pm**	**Break**	
**5:30 pm - 6:30 pm**	**Session 2 - Talks with Q&A**<br><br>**Learning from Leaders:** a dive into the resources available to help you level up your skills.	Maria Dutarte<br>Christine Broderick<br>Rebecca Mbewe<br>Linda Hunter<br>Trudy Flynn<br><br>**Moderator:** Amy Price

PxP 2023

DAY 1 CONTINUED

Time (UTC)	Session	Speakers
**6:30 pm - 7:00 pm**	**Break**	
**7:00 pm - 7:55 pm**	**Session 3 - Short Talks with Panel**<br><br>**Mentoring and being mentored:** discover support available for patients, by patients.	Janice Tufte<br>Laurie Proulx<br>Anna Samson<br>Amy Price<br><br>**Moderator**: Eileen Davidson
**7:55 pm - 8:00 pm**	**Day 1 Wrap Up**	Amy Price

PxP 2023

DAY 2: TRAILBLAZE NEW APPROACHES

Advancing health research innovation with patient partners or as a patient partner.

September 13 (UTC)

Time (UTC)	Session	Speakers
**4:00 pm - 4:10 pm**	**Day 2 Welcome**	Day two host:<br>Linda Hunter
**4:10 pm - 5:00 pm**	**Session 1 - Panel**<br><br>**Leveraging the researcher perspective:** gain insight into the mind-sets of researchers who are embracing patient partnerships.	Nader Ghasemlou<br>Linda Li<br>Diarmuid Denneny<br><br>**Moderator:** Joletta Belton
**5:00 pm - 5:30 pm**	**Break**	
**5:30 pm - 6:30 pm**	**Session 2 - Discussion**<br><br>**Taking an alternative path in (self) research:** a discussion with case studies and examples about responsible self-research and other non-traditional pathways to do research as a patient.	Dana Lewis<br>Alex Haagaard<br>Sara Riggare<br><br>**Moderator**: Trudy Flynn

PxP 2023

DAY 2 CONTINUED

Time (UTC)	Session	Speakers
**6:30 pm - 7:00 pm**	**Break**	
**7:00 pm - 7:55 pm**	**Session 3 - Talks with Q&A**<br><br>**Leading the way as a patient partner in research:** examples of and tips and resources for patient-led research.	Hannah Wei<br>Sneha Dave<br><br>**Moderator**: Linda Hunter
**7:55 pm - 8:00 pm**	**Day 2 Wrap Up**	Linda Hunter

PxP 2023

DAY 3: STRENGTHEN PATIENT PARTNERSHIPS

Raising the benchmarks for best practice.

September 14-15 (UTC)

Time (UTC)	Session	Speakers
**10:00 pm - 10:10 pm**	**Day 3 Welcome**	Day three host: Joletta Belton
**10:10 pm - 11:00 pm**	**Session 1 - Discussion**<br><br>**Facing up to the challenges:** honest discussions about overcoming real-world risks for patient partners.	Lynn Laidlaw<br>Sabrina Poirier<br>Patrick Gee<br><br>**Moderator:** Janelle Bowden
**11:00 pm - 11:30 pm**	**Break**	
**11:30 pm - 12:30 am**	**Session 2 - Talks with Q&A**<br><br>**Including all voices:** Examples and advice for doing better when working with communities historically excluded from health research.	Sandra Jayacodi<br>Alma McCormick<br>Hem Devan<br>Cheryl Davies<br><br>**Moderator:** Joletta Belton

PxP 2023

DAY 3 CONTINUED

Time (UTC)	Session	Speakers
**12:30 am - 1:00 am**	**Break**	
**1:00 am - 1:55 am**	**Session 3 - Short Talks with Panel**<br><br>**Amplifying outputs and impact of research:** how patient partners can get involved in communicating research findings.	Eileen Davidson<br>Karen Woolley<br>Joletta Belton<br><br>**Moderator**: Janice Tufte
**1:55 am - 2:00 am**	**Conference Closing Remarks**	Joletta Belton
