# Supplementary material for: Co-creating and hosting PxP: a conference about patient engagement in research for and by patient partners
Source: Res Involv Engagem. 2024 Jul 29;10:77. doi: 10.1186/s40900-024-00603-0 (PMC11287933; doi:10.1186/s40900-024-00603-0)
Supplement: Supplementary file 3 — Additional file 3. Zoom Webinar Guide that was created for conference attendees to make the most of attending the conference on the Zoom Webinar platform. [file 40900_2024_603_MOESM3_ESM.pdf]

PxP 2023 Attendee Guide for Zoom Webinars

Thank you for joining us at PxP 2023! We are looking forward to connecting with you all virtually then.

This guide is designed to show you some features on the Zoom Webinars platform to help you get the most out of the event. If you have any questions, please do not hesitate to reach out: contact@pxphub.org

This guide includes information on:

- Interacting as an attendee (p1)
- Instructions for the Chat (p2), Q&A (p3) and Reactions (p4)
- Information about turning on/off Closed Captions (p5)
- Information on Translated Captions (p5)
- Instructions for adjusting caption size/colour (p6), increasing the font size in the Chat box (p7), and adjusting screen-reader options (p7)
- The main screen views you will see during the event (p7)
- Issues with audio (p8)
- Other resources and information for Zoom Webinars (p8)

Interacting and getting involved

We would love our virtual conference to be as interactive as possible. The information below explains some of the ways that you can get involved. More detailed instructions for each of these are included further down in this guide.

1. You will be joining Zoom Webinars as an **attendee.** This means that other people joining the call will **not** be able to see or hear you. However, you will be able to see the video and sound for our event panelists and speakers.
2. We encourage you to use the **Chat** feature to join in conversations with other event attendees or to request **technical support.**
3. The **Q&A** box is for questions to the event speakers.
4. You can share **reactions** in real-time using a range of emojis on the bottom menu.
5. You can share information from the sessions on social media using #PxP23. You can find us on Twitter, LinkedIn and Instagram.

Chat

- Please use the Chat feature to join in conversations with other attendees, or request Technical Support from our team.
- To open the **Chat** window, please select the speech bubble icon on the bottom menu. This is usually towards the left of the options (orange circle on image below)

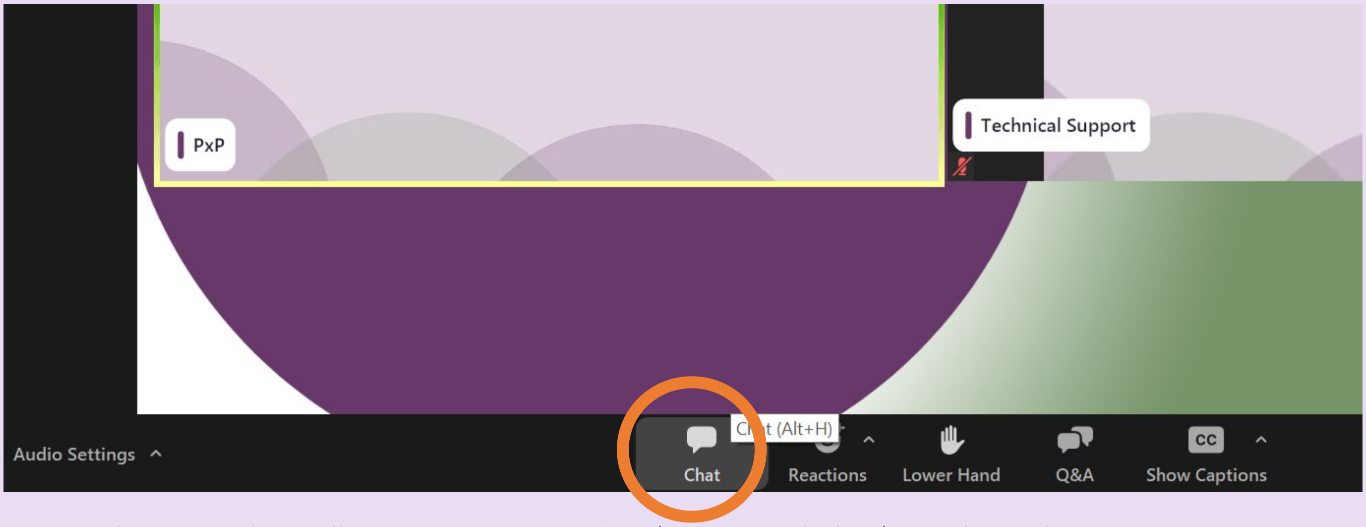

- The event chat will open in a new window (see image below). You have the option to message **Everyone** on the call (including all other event attendees), or you can message the **Hosts and panelists** only. You can switch between these options by clicking on the blue box with the small down arrow (circled in orange).
- To send a message, type in the box and press the **enter key (or return)** on your keyboard or mobile device.
- For technical support, you may find it easier to email our team contact@pxphub.org. Otherwise, you can send a message directly through Zoom chat to 'Hosts and panelists' with details of your query. A member of our team will get back to you as soon as possible.

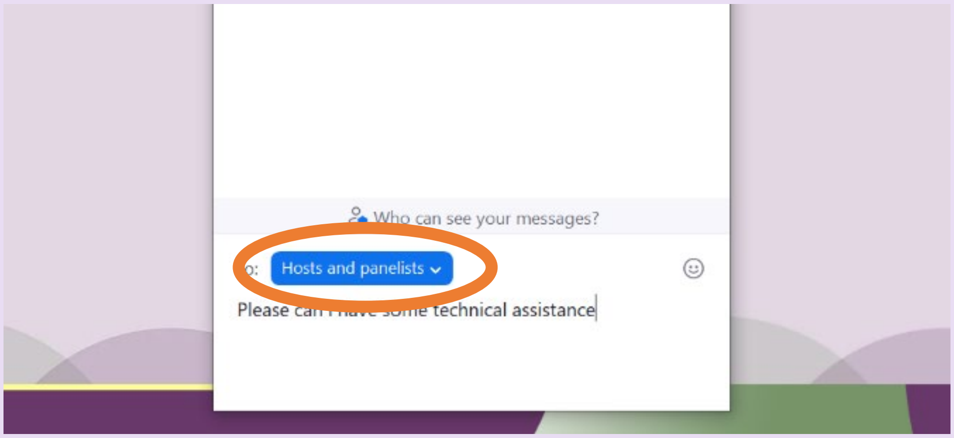

Q&A

- Please use the **Q&A** feature to ask our speakers questions during their talks, or Q&A discussions.
- Click on the Q&A icon (two speech bubbles) on the bottom menu in Zoom (orange circle on image below).

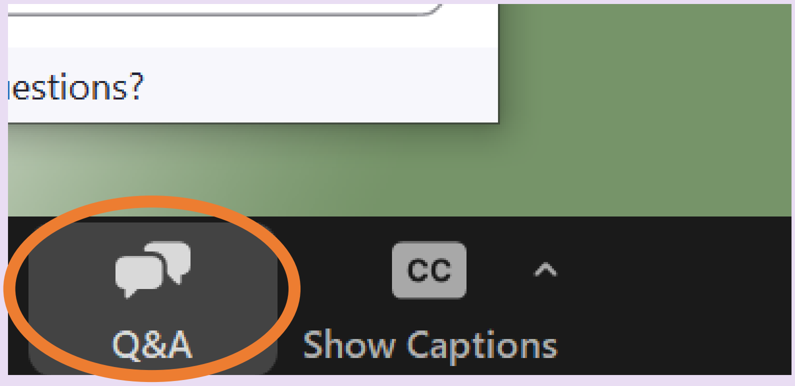

- The Q&A box will open in the middle of your screen.
- To send a question, type into the box which reads 'Type your question here...' then click on send in the bottom right.
- If you would like to send a question **anonymously**, please toggle the check-box to blue (demonstrated by the orange circle below).
- You can also **like** other people's questions (demonstrated by the red star below) or **comment** on them (demonstrated by the blue square below). Questions that receive a lot of likes may be prioritized for verbal responses during the session.
- Your question may be answered verbally, or you may receive a typed response.

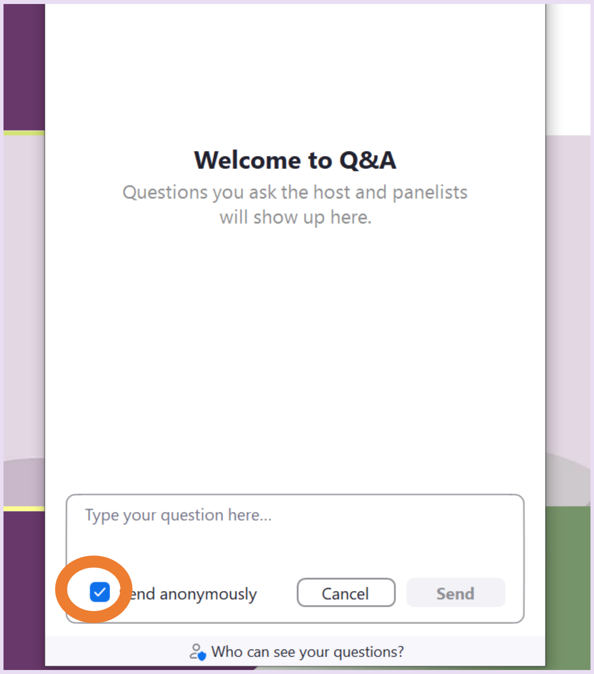

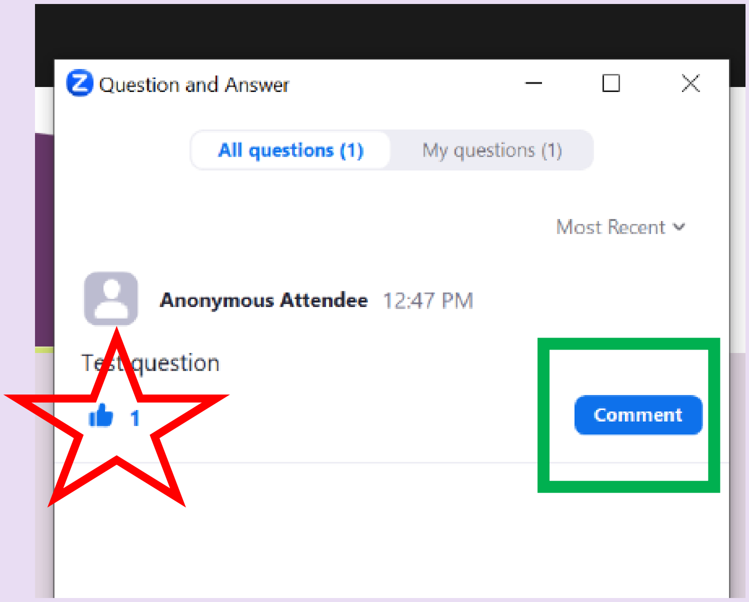

Reactions

- We encourage use of **Reactions** during the event. This is a great way to share support for the event speakers, or to let them know how you feel about a particular talk. These can be found on the bottom Zoom menu and look like a series of emojis.
- Click on the Reactions tab, and select your emoji of choice from the pop-up menu that will appear above.

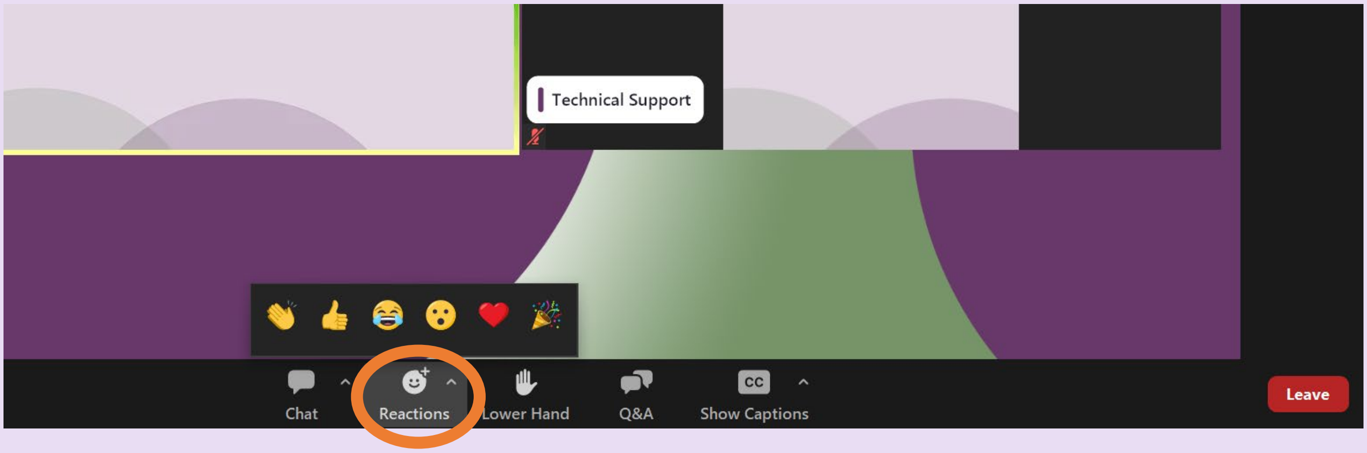

Captions and Translated Captions

- Closed Captions and Translated Captions can be accessed on the bottom menu in Zoom.
- To turn on Closed Captions, please click on the CC icon (orange circle below).
- To adjust settings, please click on the small up arrow next to the CC icon.
- This will open a menu, including a list of 36 languages which can be accessed live.

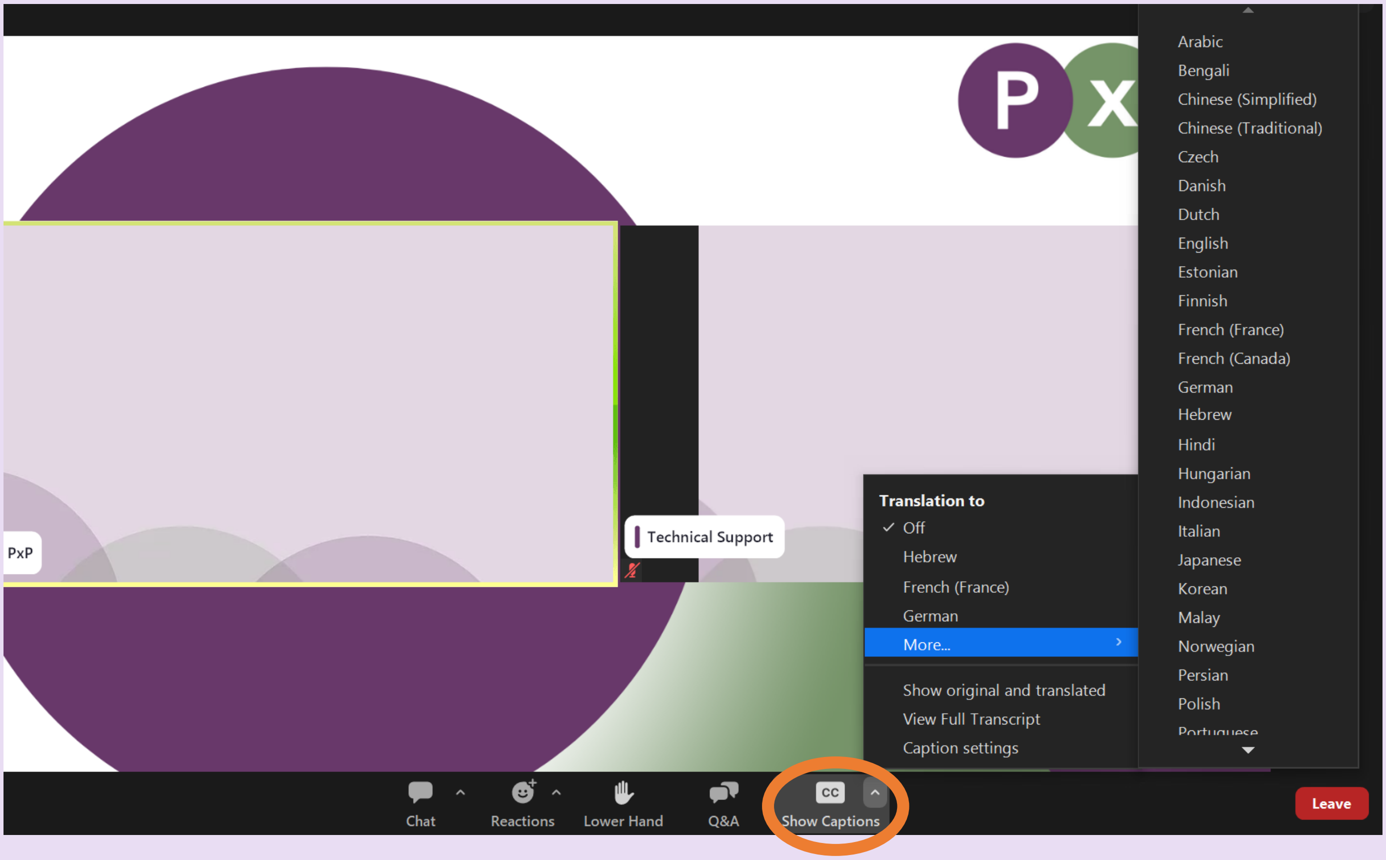

Adjust Caption Settings

- To adjust Caption Settings (font size and colour), please click on the CC icon on the bottom menu and then select **Caption Settings** (indicated by orange circle below).

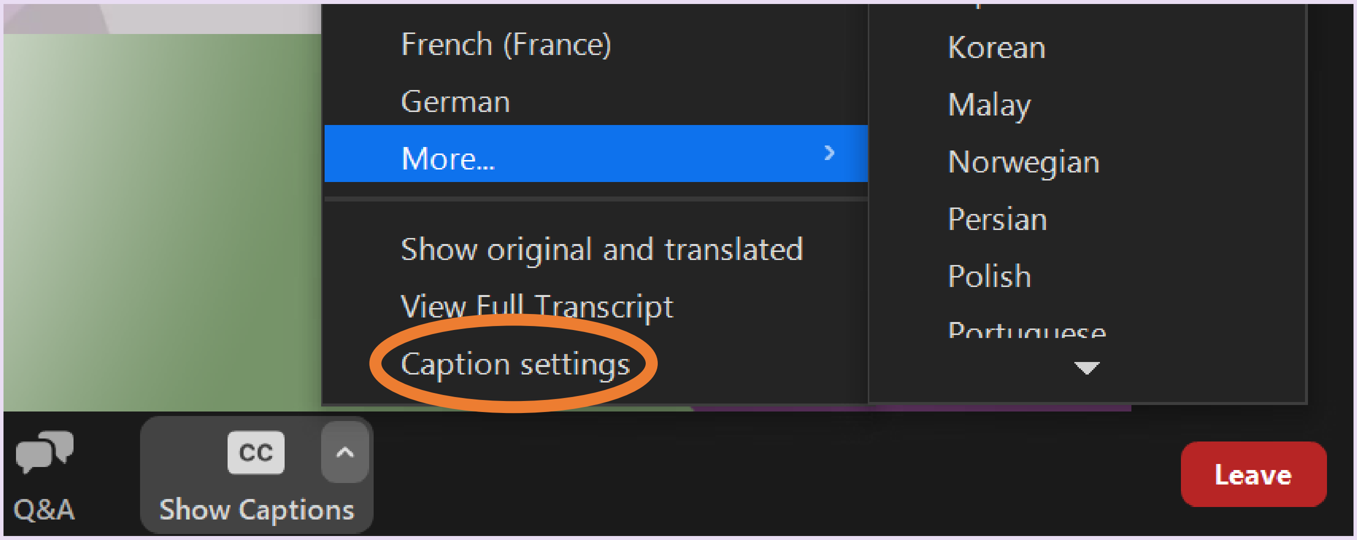

- A window will open which will allow you to adjust the font size and colour of captions, and translated captions.

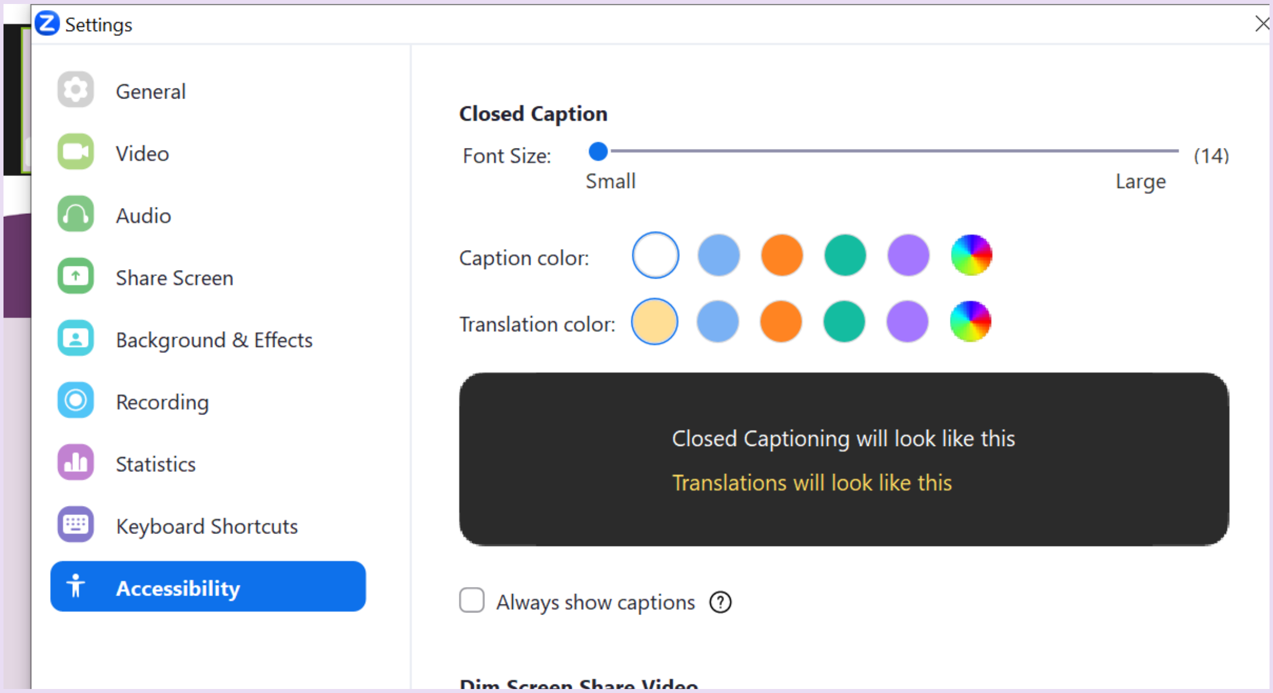

- This window can also be used to adjust the font size of the **Chat** feature (orange circle), change **Screen Reader Alerts** (blue arrow) and **Dim Screen Share Video** (Green Square).

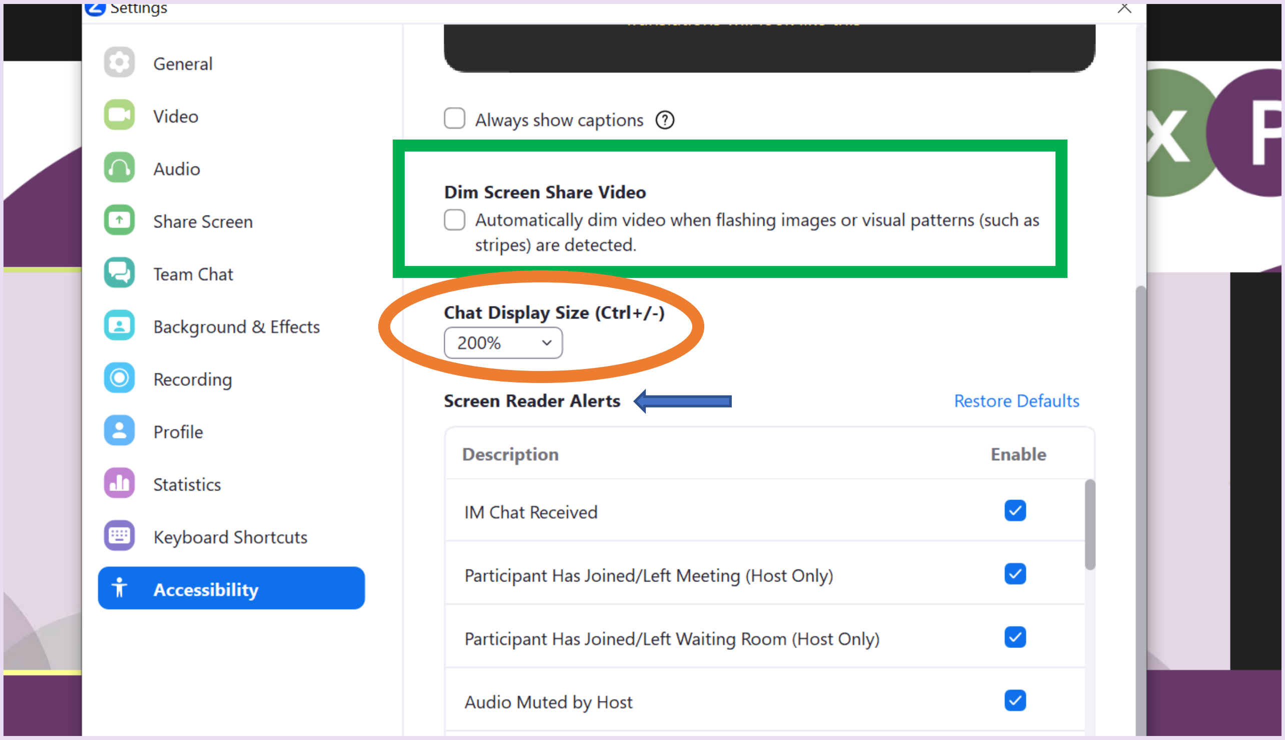

Event Views

- The pictures below show the **two** viewing options you will see during the event. These will change **automatically** during the program to try and give you the best viewing experience depending on what is happening at that moment. You will **not** need to adjust these settings yourself.
- During talks and presentations, you will see a large version of that person or their presentation in the center of your screen (Left Image). During panels, discussions and Q&A sessions, you will see the videos for multiple panelists as a kind of 'grid' (Right Image).

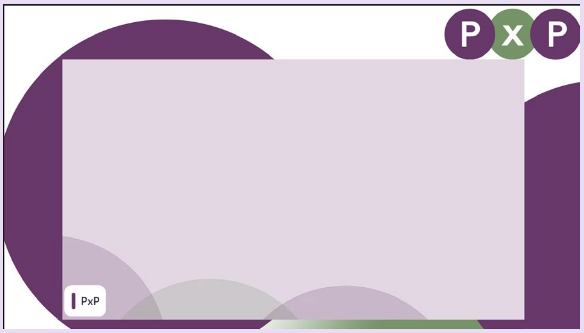

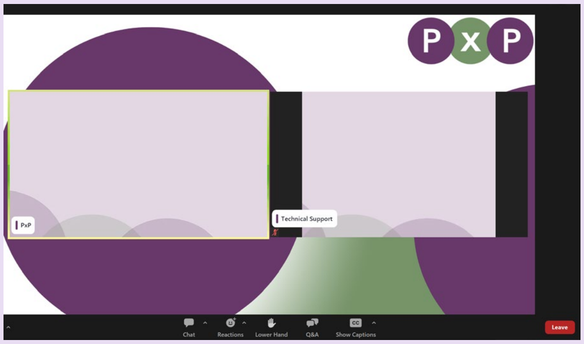

Issues with audio

If there are problems with the event sound, here are a few things you can try:

- If there is no sound coming through your speakers, check that the 'mute' button on your computer keyboard is not turned on. If you are joining from a mobile or tablet, the location of the sound controls will vary but can often be found on the side. Try turning up the volume.
- You can also find **Audio Settings** for Zoom in the bottom left hand corner of the window.

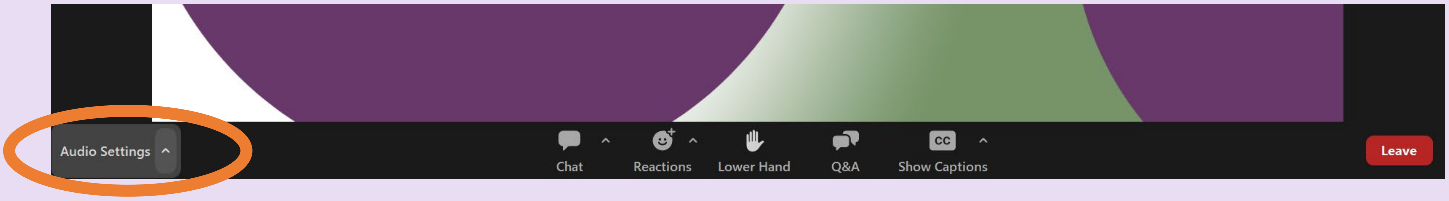

- Clicking on Audio Settings will open the window you can see in the image below. Make sure that the correct Speaker option is selected from the menu. You can also try testing the speaker using this menu.
- You could also try using headphones if these are comfortable for you, and you have a pair to hand.

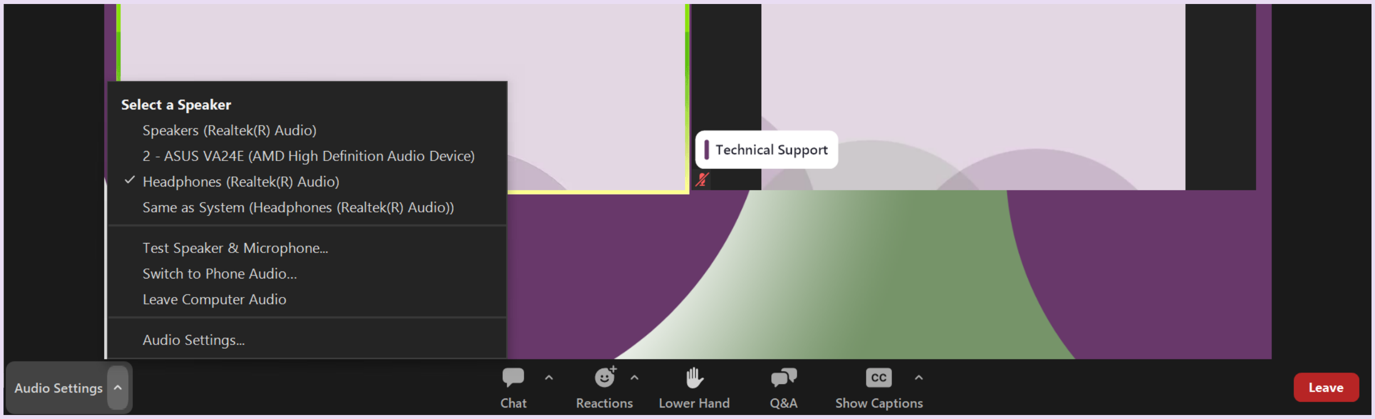

Other Useful Resources for Zoom Webinars

Downloading Zoom: https://support.zoom.us/hc/en-us/articles/201362233
Note: A Zoom account is **not** needed to join the PxP webinars. You can join as a participant without creating an account. However, you will need to download the Zoom software.

Joining a webinar:
https://support.zoom.us/hc/en-us/articles/115004954946-Joining-and-participating-in-Zoom-Webinars

Sound issues on your mobile device:
https://support.zoom.us/hc/en-us/articles/204484835-My-Audio-is-Not-Working-on-iOS-or-Android
